# Implementation of p16/Ki67 dual stain cytology in a Danish routine screening laboratory: Importance of adequate training and experience

**DOI:** 10.1002/cam4.3399

**Published:** 2020-09-07

**Authors:** Anne Hammer, Line W. Gustafson, Pia N. Christensen, Rikke Brøndum, Berit Andersen, Rikke H. Andersen, Mette Tranberg

**Affiliations:** ^1^ Department of Obstetrics and Gynecology Regional Hospital West Jutland Herning Denmark; ^2^ Department of Clinical Medicine Aarhus University Aarhus Denmark; ^3^ Department of Public Health Programmes Randers Regional Hospital Randers Denmark; ^4^ Department of Pathology Randers Regional Hospital Randers Denmark

**Keywords:** cervical cancer screening, cytology, dual stain, implementation, mass screening, training

## Abstract

**Background:**

Immunocytochemical staining with p16/Ki67 has been suggested as a promising triage biomarker in cervical cancer screening. As dual staining is a subjective method, proper training may be required to ensure safe implementation in routine laboratories and reduce risk of misclassification. We determined concordance between novice evaluators and an expert, stratified by number of slides reviewed at three reading points.

**Methods:**

The study was conducted at the Department of Pathology, Randers, Denmark. Women were eligible if they were aged ≥45, had been enrolled in one of two ongoing clinical studies, and had a dual stain slide available. Dual staining was performed using the CINtec plus assay. Slides were randomly selected from three reading points at which novice evaluators had reviewed <30, ~300, and ≥500 dual stain slides respectively. Level of concordance was estimated using Cohen's Kappa, *κ*.

**Results:**

Of 600 eligible slides, 50 slides were selected for review as recommended by the manufacturer. Median age was 68 years (range: 58‐74). Overall concordance was good (*κ* = 0.68, 95% confidence interval [CI]: 0.60‐0.76), with an overall agreement of 84% (95% CI: 70.9%‐92.8%). Concordance improved with increasing number of slides reviewed at a given reading point, from a moderate concordance (*κ* = 0.47, 95% CI: 0.05‐0.90) after reviewing <30 slides to a good concordance (*κ* = 0.66, 95% CI: 0.20‐0.88) and a very good concordance (*κ* = 0.88, 95% CI: 0.66‐1.00) after reviewing ~300 and ≥500 slides, respectively.

**Conclusions:**

When interpreting dual stain slides from older women, concordance increased slightly as novice evaluators received more training and experience. Although further evaluation is warranted, these findings indicate that a significant amount of training and experience of novice evaluators may be needed to ensure accurate dual stain interpretation in this age group. Future studies should accurately describe training and experience of evaluators to enable a better comparison of concordance and diagnostic accuracy across studies.

**Trial registration:**

NCT04114968 and NCT04298957.

## Graphical abstract

As training and experience in p16/Ki67 dual stain interpretation may affect concordance and possibly diagnostic accuracy of cervical precancer, it is important that future studies accurately describe any training and previous experience among evaluators to allow for a meaningful comparison of dual stain accuracy across studies. Furthermore, setting up proper dual stain training programs in routine screening laboratories is advisable to ensure sufficient interpretation skills among novice evaluators.

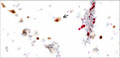

## 1 INTRODUCTION

High‐risk human papillomavirus (HPV) testing is more sensitive for detecting cervical intraepithelial neoplasia grade 3 or worse (CIN3+) compared to cytology.^1^, ^2^ As a result, HPV testing is gradually replacing cytology in cervical cancer screening programs in many developed countries. Because most HPV infections are transient, known to carry a low risk of CIN3+, triage testing of HPV‐positive is needed to avoid unnecessary colposcopies and biopsies. Dual immunocytochemical staining for p16/Ki67 with the commercial CINtec^®^ PLUS assay has been proven to be a sensitive and specific triage test for HPV‐positive women^3^, ^4^, ^5^, ^6^ as well as for women with low‐grade cytological abnormalities,^7^, ^8^ and may provide greater reassurance against CIN3+^9^ than cytology alone. Compared to molecular‐based triage tests, the interpretation of dual staining is evaluator‐dependent and not automated. Consequently, proper training of evaluators may be critical to achieve safe implementation of dual staining in routine screening laboratories with little or no previous experience in dual stain interpretation, and to reduce risk of misclassification. Yet, some studies state that implementation of p16/Ki67 dual staining is feasible in routine screening laboratories with minimal training of evaluators.^10^, ^11^ At present, there is no consensus on what defines adequate training.^12^ Most^3^, ^6^, ^9^, ^13^, ^14^, ^15^, ^16^ but not all studies^11^ are lacking detailed information about the training program provided to evaluators prior to study start and their level of experience with dual stain interpretation, including whether evaluators had any previous experience with cytology interpretation. This may challenge a meaningful comparison of dual stain accuracy and clinical performance across studies.

Whereas most previous studies have focused on women in the screening age, only one study has explored the value of dual stain triage among postmenopausal women (≥50 years).^17^ However, because of low cellularity and cellular atrophy, dual stain interpretation may be more challenging among older postmenopausal women. Thus, more training and experience may be required to achieve and sustain an acceptable concordance and diagnostic accuracy.

Here, we describe our experience with implementation of p16/Ki67 dual stain for triage of older women with abnormal screening results in a Danish routine screening laboratory. More specifically, we describe the concordance in dual stain results between an expert in dual stain interpretation and novice evaluators, overall and stratified by the number of slides interpreted. Additionally, we propose ideas for setting up a suitable training program for novice evaluators.

## 2 METHODS

### 2.1 Setting

This study was conducted at the Department of Pathology, Randers Regional Hospital, Denmark, which is responsible for processing all cervical cytology samples obtained in Central Denmark Region (ie 85, 000‐100, 000 samples annually). In Denmark, cervical cancer screening is organized and free of charge. At present, women aged 23‐59 years are screened with cytology, whereas women aged 60‐64 years undergo HPV‐based screening. At the department, cytology slides are interpreted by cytotechnicians using computer‐assisted microscopy (FocalPoint™ GS Imaging System; BD Diagnostics) and categorized according to the Bethesda 2014 grading system.^18^ Human papillomavirus DNA testing is performed using the Cobas 4800 platform (Roche Diagnostics). To ensure high quality of cytology screening, all cytotechnicians in Denmark are recommended, but not required, to pass the Quality Assurance, Training, and Examinations Committee (QUATE) exam provided by European Federation of Cytology Societies (EFCS).^19^, ^20^ Currently, dual staining is not used routinely within the Danish cervical cancer screening program.

#### 2.1.1 Population

Slides for this concordance analysis were selected from two ongoing clinical studies in Denmark evaluating the accuracy and clinical performance of p16/Ki67 dual staining among women aged 65‐69 years testing HPV positive as part of an additional screening offer (NCT04114968), and women aged 45 years and older referred to colposcopy due to an abnormal screening result (threshold: ≥atypical squamous cell of undetermined significance cytology, ASC‐US+ and/or HPV‐positive) (NCT04298957). Slides were collected in the period from March 2019 through December 2019. At the time of the present study, histological results were not available.

### 2.2 Slide preparation and p16/Ki67 dual staining

Slides for p16/Ki67 dual stain testing were produced from the residual cell‐pellet material of the liquid‐based Surepath™ cytology samples and prepared using the Totalys^TM^ Slide‐Prep (BD Diagnostics). Subsequently, slides were stained using the Conformité Européene‐In Vitro Diagnostics and Federal Drug Agency‐approved CINtec^®^ PLUS assay (Roche Diagnostics)^21^ and the automated BenchMark ULTRA immunostainer (VENTANA; Roche Diagnostics) according to the manufacturer's instructions. After immunostaining, aqueous mounting media (CC/Mount™) was applied to slides, and after drying overnight slides were incubated in xylene and mounted with regular coverslip. Each staining run included one external positive control (high‐grade squamous intraepithelial lesion sample). Cells stained with only p16 and Ki67 were used an internal positive control.

### 2.3 Dual stain interpretation

**FIGURE 1:**
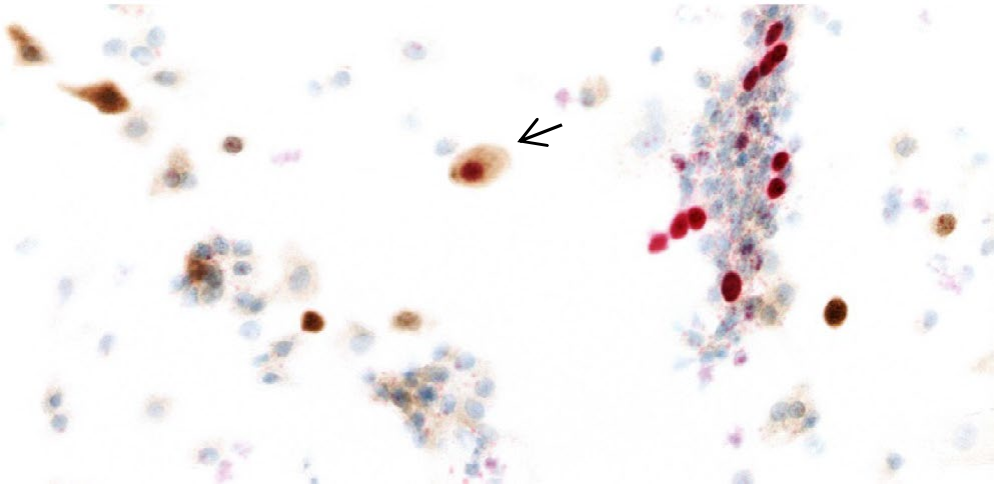
The image illustrates one dual‐stain positive cell. Objective: X20

Two experienced, QUATE‐certified cytotechnicians with more than 10 years of experience independently evaluated dual stained slides using a standard microscope. The cytotechnicians were blinded to all study data (ie HPV genotype, cytology and histology results), except age of the woman. In case of disagreement between the two cytotechnicians, a consensus score was reached and considered definitive. The cytotechnicians also recorded information on staining characteristics, such as staining intensity, background staining, and counter staining. A dual stain slide was considered positive when at least one dual‐stain positive cell was identified (ie cytoplasmic brown staining for p16 and nuclear red staining for Ki67 in the same cervical epithelial cell), without consideration of morphology and cellularity criteria (Figure 1). Slides were deemed negative if they met the squamous cellularity criteria (ie ≥5000 cells per slide as specified in the Bethesda 2014 criteria) and contained no dual‐stain positive cells. Negative slides that did not meet the cellularity criteria and slides with no staining for one of the two proteins were considered inadequate. In this case, a second slide was prepared if residual material was available.

### 2.4 Training program

Both cytotechnicians had no previous experience with preparation and interpretation of dual stained slides. Therefore, two 1‐day training courses were provided by the manufacturer and conducted by an expert in dual stain interpretation. The first course took place in May 2019 and included lectures of the interpretation guide. The course ended with an exam of 40 teaching slides provided by the manufacturer, with a passing grade of 90%. Both cytotechnicians passed the exam. In August 2019, cytotechnicians started evaluating dual stained slides. A second and identical training course, led by the same expert, took place in December 2019. At this course, patient slides considered difficult to interpret and the consensus‐based slides were reviewed and discussed with the expert using a multiheaded microscope.

### 2.5 Concordance design

After completing the training courses, the manufacturer recommended an agreement score of ≥90% between cytotechnicians and the expert when comparing dual stain results of 50 random patient slides. The manufacturer provided no specified demands or recommendations for selection of slides. Assuming that the number of slides evaluated by the cytotechnicians at a given time could influence the agreement score, we chose to randomly select slides from three different reading points between August 2019 and December 2019 as presented in Table 1. In January 2020, the 50 random slides were reviewed by the expert who was blinded to the cytotechnicians' results and other study data.

**TABLE 1:** Design of the concordance assessment

Reading point	Slide number	Time of evaluation	Number of dual‐stained slides reviewed by cytotechnicians
1	1‐16	Early August 2019	<30
2	17‐34	Mid‐September 2019	~300
3	35‐50	Mid‐December 2019	≥500

### 2.6 Statistical analyses

Concordance in dual stain results between cytotechnicians and the expert was assessed using Kappa statistics (Cohen's Kappa, *κ*), including 95% confidence intervals (CIs), and defined as "Poor" (*κ* ≤ 0.20), "Fair" (0.21 ≤ *κ* ≤ 0.40), "Moderate" (0.41 ≤ *κ* ≤ 0.60), "Good" (0.61 ≤ *κ* ≤ 0.80), or "Very good" (*κ* ≥ 0.81).^22^, ^23^ The overall percentage agreement with corresponding 95% CI was calculated as the proportion of concordant slides divided by the total number of slides, overall and stratified by number of slides evaluated. Cochrane‐Armitage test was used to test for trends in agreement rates; reading point 1 vs reading point 2 vs reading point 3. Dual stain results reported by the expert was considered as the reference for comparison. All data were double‐entered and stored in REDCap.^24^, ^25^ *P* < .05 were considered statistically significant. All statistical analyses were conducted using STATA version 16 (StataCorp LP).

## 3 RESULTS

**FIGURE 2:**
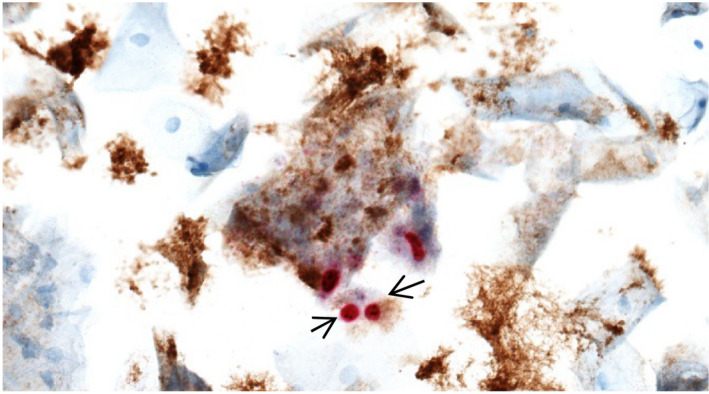
Staining characteristics of discordant cases. Arrows point to single dual‐stain positive cells. A strong background staining makes it difficult to determine whether the slide should be scored positive or not. Objective: X20

A total of 600 slides from the two studies were eligible for selection and, as recommended by the manufacturer, 50 slides were randomly selected for evaluation. Forty‐five slides were from women aged 65‐69 undergoing primary HPV screening and five slides were from women ≥45 years referred to colposcopy. Median age of women whose slides were included in the present analysis was 68 years (range: 58‐74 years). For the 50 slides, the two cytotechnicians reported identical results. Concordance in dual stain results between cytotechnicians and the expert was good (*κ* = 0.68, 95% CI: 0.60‐0.76), with an overall agreement of 84.0% (95% CI: 70.9%‐92.8%) (Table 2). A total of eight slides (16.0%) had disconcordant dual stain results. In four cases, slides were scored expert negative/cytotechnician positive. In two of these cases the discordance might be explained by strong background staining, making it difficult to determine whether a cell was positive or not (Figure 2). Three slides were scored expert positive/cytotechnician negative. Of these, one case had only one dual‐stain positive cell, which was missed by both cytotechnicians. One slide was scored inadequate by the expert and negative by the cytotechnicians (Table 2). Concordance and overall agreement in dual stain results, stratified by number of slides reviewed at a given reading point are provided in Table 3. At the first reading point (<30 slides reviewed), concordance in dual stain results between cytotechnicians and the expert was moderate (*κ* = 0.47, 95% CI: 0.05‐0.90) and improved to good (*κ* = 0.66, 95% CI: 0.20‐0.88) at the second reading point (∼300 slides reviewed) and to very good (*κ* = 0.88, 95% CI: 0.66‐1.00) at the third reading point (≥500 slides reviewed) (Table 3). The percentage agreement in dual stain results was 75.0% (95% CI: 47.6%‐92.7%) at the first reading point and increased to 82.4% (95% CI: 56.6%‐96.2%) and 94.1% (95% CI: 71.3%‐99.9%) at the second and third reading points, respectively. However, the increase between the three reading points was not statistically significant (*P* = .13). Results remained robust after restricting our analysis to slides that were deemed adequate by all evaluators (n = 49).

**TABLE 2:** Concordance and agreement in dual stain results of 50 randomly selected slides

All 50 slides	Expert (reference)								*κ* ^a^ (95% CI)	Agreement (%) (95% CI)
	Positive		Negative		Unsatisfactory		Total			
	n	%^b^	n	%	n	%	n	%		
Cytotechnicians										
Positive	18	36.0	4	8.0	1	2.0	23	46.0		
Negative	3	6.0	24	48.0	0	0.0	27	54.0	0.68 (0.60‐0.76)	84.0 (70.9‐92.8)
Unsatisfactory	0	0.0	0	0.0	0	0.0	0	0.0		
Total	21	42.0	28	56.0	1	2.0	50	100.0		

^a^ Cohens Kappa. "Poor" (*κ* ≤ 0.20), "fair" (0.21 ≤ *κ* ≤ 0.40), "moderate" (0.41 ≤ *κ* ≤ 0.60), "good" (0.61 ≤ *κ* ≤ 0.80), or "very good" (*κ* ≥ 0.81).^22^

^b^ % = Row percentage.

**TABLE 3:** Concordance and agreement in dual stain results between cytotechnicians and the expert, stratified by the number of slides reviewed

	Expert (reference)								*κ* ^a^ (95% CI)	Agreement (%) (95% CI)
	Positive		Negative		Unsatisfactory		Total			
	n	%^b^	n	%	n	%	n	%		
First reading point (<30 slides reviewed)										
Cytotechnicians										
Positive	4	25.0	3	18.8	0	0.0	7	43.8	0.47 (0.05‐0.90)	75.0 (47.6‐92.7)
Negative	1	6.3	8	50.0	0	0.0	9	56.3		
Unsatisfactory	0	0.0	0	0.0	0	0.0	0	0.0		
Total	5	31.3	11	68.8	0	0.0	16	100.0		
Second reading point (~300 slides reviewed)										
Cytotechnicians										
Positive	6	35.3	1	5.9	1	5.9	8	47.1	0.66 (0.20‐0.88)	82.4 (56.6‐96.2)
Negative	1	5.9	8	47.1	0	0.0	9	52.9		
Unsatisfactory	0	0.0	0	0.0	0	0.0	0	0.0		
Total	7	41.2	9	52.9	1	5.9	17	100.0		
Third reading point (≥500 slides reviewed)										
Cytotechnicians										
Positive	8	47.1	0	0.0	0	0.0	8	47.1	0.88 (0.66‐1.00)	94.1 (71.3‐99.9)
Negative	1	5.9	8	47.1	0	0.0	9	52.9		
Unsatisfactory	0	0.0	0	0.0	0	0.0	0	0.0		
Total	9	52.9	8	47.1	0	0.0	17	100.0		

^a^ Cohens Kappa. "Poor" (*κ* ≤ 0.20), "fair" (0.21 ≤ *κ* ≤ 0.40), "moderate" (0.41 ≤ *κ* ≤ 0.60), "good" (0.61 ≤ *κ* ≤ 0.80), or "very good" (*κ* ≥ 0.81).^22^

^b^ %=Row percentage.

## 4 DISCUSSION

### 4.1 Main findings

In the present study, we found an overall good concordance in p16/Ki67 dual stain interpretation between an expert and two novice evaluators (*κ* = 0.68), with an agreement score slightly below the recommended score set by the manufacturer (84% vs ≥90%). The level of concordance tended to increase with increasing number of slides interpreted by the novice evaluators, from moderate concordance after reviewing <30 slides to very good concordance after reviewing more than 500 slides from women aged 45 years and older. These concordance results may demonstrate that a significant amount of training and experience of novice evaluators may be needed to ensure accurate dual stain interpretation, particularly when evaluating slides from older women.

### 4.2 Interpretation and comparison with other studies

Our results are in line with some studies reporting a good concordance in dual stain interpretation between new and experienced evaluators, ranging from a kappa value of 0.61‐0.70.^10^, ^26^ Another study reported a moderate concordance (*κ* = 0.49),^16^ while two studies reported a very good concordance (*κ* = 0.82‐0.89).^11^, ^27^ The underlying reason for these differences across studies is unclear, but may be explained by differences in characteristics of the study cohort, including age of the women, and whether p16/Ki67 was performed as triage of HPV‐positive women, among women with abnormal cytology, or in a population of women referred to colposcopy. Additionally, training and level of experience with dual stain and cytology interpretation among evaluators prior to study start may differ between studies. Our study suggests that a significant amount of training may be needed to achieve an agreement of ≥90%, as recommended by the manufacturer. Agreement exceeded the recommended threshold after attending a second training course and after reviewing more than 500 slides, a number that is well above that reviewed by evaluators in most previous studies assessing interobserver variation.^11^, ^12^, ^26^, ^27^ This may be due to the fact that we, as opposed to most previous studies, only included slides from older women that may be more difficult to interpret because of cellular atrophy. To the best of our knowledge, only one study has demonstrated that dual stain testing may be useful for triage in postmenopausal women diagnosed with low‐grade cytological abnormalities,^17^ but none have explored level of concordance in dual stain interpretation among this older age group. Nevertheless, our findings are in agreement with other studies in which additional training with dual stain interpretation resulted in increased concordance,^10^, ^12^, ^16^, ^28^ with a higher concordance among evaluators experienced in dual stain interpretation as compared to new evaluators (*κ* = 0.74 vs *κ* = 0.50).^26^ Additionally, whereas some studies defined experts as evaluators with great experience in dual stain interpretation,^11^ other studies defined experts as evaluators with great experience in cytology interpretation^10^ or p16 single immunostaining.^27^ These differences in how an “expert” was defined may have affected level of concordance across studies, particularly because the “expert” is typically used as a reference. Together, these findings may suggest that proper training is needed to reach the recommended level of agreement and that continued training and/or supervision may be needed to sustain an acceptable concordance.

In the present study, discordance in dual stain results was mostly due to slides being categorized as positive by novice evaluators, but deemed negative by the expert, a finding also replicated by others.^27^ In some cases, we found that the disagreement was explained by strong background staining making it difficult to decide if the cell should be scored dual stain positive, an issue raised previously.^12^

As the main goal of triage testing in cervical cancer screening is to improve specificity while maintaining a high sensitivity, it is important to assess whether training and experience of evaluators might affect the test's clinical performance. This will be particularly important when implementing p16/Ki67 dual stain cytology in routine screening laboratories across the globe, with no or minimal experience in dual stain interpretation. To our knowledge only a few studies have sought to evaluate clinical performance among experienced and new evaluators. Two studies reported no meaningful difference in sensitivity and specificity for CIN2 + detection between new and experienced evaluators,^11^, ^26^ indicating that p16/Ki67 may easily be implemented in routine screening laboratories, whereas another study reported ambiguous findings.^12^ Thus, continued surveillance of p16/Ki67 dual stain accuracy will be important following the implementation of p16/Ki67 in routine screening laboratories.

Although most studies report p16/Ki67 dual stain to have almost similar sensitivity as cytology for detecting CIN3+ and significantly better specificity,^4^, ^13^, ^15^ some studies report dual stain to be more sensitive compared to cytology, with nearly identical specificity.^6^, ^14^ Indeed, there may be many explanations for these discrepancies, such as differences between study population included (ie HPV positive women, ASC‐US+ women, women referred to colposcopy, etc) and the quality of cytology screening in the setting. Unfortunately, most^6^, ^10^, ^13^, ^14^, ^15^, ^26^ but not all^11^ previous studies include no detailed information on training received prior to study start, including detailed information about previous experience with dual stain and/or cytology interpretation. Thus, without more information on training and previous experience of evaluators it remains unclear whether these ambiguous results to some extent may be explained by differences in training and previous experience with dual stain interpretation.

### 4.3 Training program and quality assurance

Monitoring interpretation skills of novice evaluators might be a key element in the training program to ensure safe implementation of dual staining in routine laboratories. This might be done by assessing dual stain agreement rates between experts and novice evaluators using slides from different age groups with varying percentages of CIN2/CIN3 cases. Improvement (or lack thereof) in interpretation skills of evaluators could be monitored by measuring the time needed to evaluate one slide, as suggested elsewhere.^12^ Although our study was not designed to assess the value of continued expert‐led training of novice evaluators, face‐to‐face or digital work‐shops with experts may be needed to enable discussion and review of difficult cases. Comparable to cytology screening, high quality of dual stain interpretation may be secured by recommending external exams and possibly certification for those evaluating dual stain slides, similar to the QUATE exam provided by the EFCS. External quality control of the immunocytochemical staining procedure may be provided by the NordicQC or UK‐NEQAS associations.^29^, ^30^ To share knowledge on the amount of training required to achieve sufficient interpretation skills, it may be of great value to establish a network of experts and cytotechnicians from routine screening laboratories. This network might also lead to consensus of what constitutes a proper dual stain training program.^28^

### 4.4 Strengths and limitations

A key strength of the present study was the concordance design: selection of slides from three different reading points, allowing us to monitor the learning curve for novice evaluators and estimate the number of evaluated slides needed to achieve sufficient interpretation skills. Furthermore, slide evaluation was conducted blinded to other study outcomes, thereby eliminating information bias. However, important limitations should be mentioned. First, the majority of slides included in the present analysis were from older postmenopausal women, which may have affected the generalizability of study results. Cytology interpretation is often more challenging in this subgroup, particularly because of low cellularity and the presence of atrophic epithelial cells. Consequently, interpretation of dual stain results might be considered more challenging in this population compared to a screening population, possibly resulting in a lower level of concordance between expert and novice evaluators in the current study. Second, the low number of slides included in the analysis (50 slides) may be considered a limitation; however, the number of slides included was in accordance with the manufacturer's recommendation, and the training program provided by the manufacturer is assumed to be more or less the same across countries. Thus, our findings may be of importance to other routine screening laboratories that are in the process of implementing p16/Ki67 dual stain. Finally, our study was conducted using SurePath liquid‐based cytology samples, and the results might not be replicated with other cytology fixatives and conventional cytology.

In conclusion, we found an overall good concordance in dual stain interpretation of slides from older women between novice evaluators and an expert, with rising level of concordance with increasing experience in dual stain interpretation and additional training. As training and experience may affect concordance estimates and possibly diagnostic accuracy, future studies should carefully describe training and experience as this will allow for a more meaningful comparison of results across studies. Future research should also focus on reaching consensus of what constitutes a proper dual stain training program in routine screening laboratories.

## CONFLICTS OF INTERESTS

Roche Denmark has provided Cobas HPV‐DNA test kits and CINtec Plus test kits for the study at no cost. According to the contract between Roche and the Department of Public Health Programmes, Randers Regional Hospital, Denmark, the manuscript has been sent to Roche for review, but Roche had no influence on the scientific process and no editorial rights pertaining to this manuscript. The authors retained the right to submit the manuscript. AH and LWG have received a speaker's fee from Astra Zeneca, Denmark, outside of the submitted work. MT has received speaker's fees from Astra Zeneca, Denmark, and Roche Diagnostics, Denmark, outside of the submitted work. MT and BA have participated in other studies with HPV test kits sponsored by Roche and HPV self sampling devices sponsored by Axlab. The remaining authors declare no conflicts of interest.

## AUTHOR CONTRIBUTIONS

The study was designed by AH, LWG, BA, and MT. Enrollment of patients and selection of cases for review was conducted by LWG and MT. Preparation and interpretation of slides was conducted by PNC, RB, and RHA under supervision from MT. Statistical analyses were performed by MT and subsequently verified by AH, LWG, and BA. All authors participated in writing and reviewing the paper. All authors approved the final version.

## Data Availability

Restrictions apply for the availability of these data, which were used under the license of this study. Data are available from the authors with the permission from the Danish Data Protection Agency.
